# Hip, vertebral, and wrist fracture risks and schizophrenia: a nationwide longitudinal study

**DOI:** 10.1186/s12888-022-03723-7

**Published:** 2022-02-01

**Authors:** Yu-Wen Chu, Wen-Pin Chen, Albert C. Yang, Shih-Jen Tsai, Li-Yu Hu, Shyh-Chyang Lee, Yao-Tung Lee, Cheng-Che Shen

**Affiliations:** 1 Department of Pharmacy, Taichung Veterans General Hospital, Taichung, Taiwan; 2 Faculty of Pharmacy, School of Pharmaceutical Sciences, National Yang-Ming University, Taipei, Taiwan; 3 Center for General Education, Tunghai University, Taichung, Taiwan; 4 Department of Radiology, Ditmanson Medical Foundation Chia-Yi Christian Hospital, Chiayi, Taiwan; 5 Institute of Brain Science, National Yang-Ming University, Taipei, Taiwan; 6 Division of Interdisciplinary Medicine and Biotechnology, Beth Israel Deaconess Medical Center/Harvard Medical School, Boston, MA USA; 7 Department of Psychiatry, Taipei Veterans General Hospital, Taipei, Taiwan; 8 Division of Psychiatry, National Yang-Ming University, Taipei, Taiwan; 9 Department of Orthopedics, Chiayi Branch, Taichung Veterans General Hospital, Chiayi, Taiwan; 10 Department of Psychiatry, Shuang Ho Hospital, Taipei Medical University, No.291, Zhongzheng Rd., Zhonghe District, New Taipei City, 23561 Taiwan; 11 Department of Psychiatry, School of Medicine, College of Medicine, Taipei Medical University, Taipei, Taiwan; 12 Center of dementia, Shuang Ho Hospital, Taipei Medical University, New Taipei city, Taiwan; 13 Department of Psychiatry, Chiayi Branch, Taichung Veterans General Hospital, No. 600, Sec. 2, Shixian Rd., West District, Chiayi City, Taiwan; 14 Center for Innovative Research on Aging Society (CIRAS), National Chung Cheng University, Chiayi City, Taiwan

**Keywords:** Schizophrenia, Fracture, Hazard ratio, National Health Insurance Research Database, Cohort study

## Abstract

**Background:**

Fractures are a great health issue associated with morbidity, quality of life, life span, and health care expenditure. Fractures are correlated with cardiovascular disease, type 2 diabetes mellitus, cerebrovascular disease, and some psychiatric disorders. However, representative national data are few, and longitudinal cohort studies on the association between schizophrenia and the subsequent fracture risk are scant. We designed a nationwide population-based cohort study to investigate the association of schizophrenia with hip, vertebral, and wrist fractures over a 10-year follow-up.

**Methods:**

Data of patients with schizophrenia (*International Classification of Diseases, Ninth Revision, Clinical Modification* code 295) and matched over January 2000–December 2009) were extracted from Taiwan National Health Insurance Research Database. A Cox proportional-hazards regression model was constructed to calculate hazard ratios (HRs) for fractures between the schizophrenia and control cohorts.

**Results:**

Of 2028 people with schizophrenia (mean age: 36.3 years, 49.4% female), 89 (4.4%) reported newly diagnosed fractures—significantly higher than the proportion in the control population (257, 3.2%; *P* = 0.007). The incidences of hip (1.2%, *P* = 0.009) and vertebral (2.6%, *P* = 0.011) fractures were significantly higher in the schizophrenia cohort than in the control cohort. In Cox regression analysis, hip (adjusted HR: 1.78, 95% confidence interval [CI]: 1.08–2.93) and vertebral (adjusted HR: 1.40, 95% CI: 1.01–1.95) fracture risks were significantly higher in patients with schizophrenia. Furthermore, a sex-based subgroup analysis revealed that the risk of hip fracture remained significantly higher in female patients with schizophrenia (HR: 2.68, 95% CI: 1.32–5.44) than in female controls. On the other hand, there was no significant interaction between effects of sex and schizophrenia on the risk of fractures.

**Conclusions:**

Over a 10-year follow-up, hip and vertebral fracture risks were higher in the people with schizophrenia than in the controls. The risk of fractures in patients with schizophrenia does not differ between female and male.

## Background

Fractures may contribute to a series of health problems, with consequences including morbidity, reduced lifespan, worsened quality of life, and high health care costs [1]. In 2000, an estimated 9.0 million osteoporotic fractures were reported worldwide, with the top three fracture sites being the hip, forearm, and vertebrae [2]. As life expectancy increases, more people will be at risk of suffering these fractures that the prevalence is expected to reach > 2 million for hip fracture in 2020 and > 4 million for vertebral fracture in 2050 [3]. Hip fracture is one of the most prominent contributors to morbidity and mortality; moreover, health care costs increase because of increased health service use [4]. Although the trend in its mortality is changing, the mean overall mortality at 1 year after hip fracture has remained extremely high (22.0%) [5], and the survivors may require social and nursing care [6]. Spinal fracture leading to vertebral compression has exhibited a similar trend; it affects approximately 1.5 million US adults annually, and the severe deconditioning leads to considerably higher mortality in patients with spinal fracture than in age-matched controls [7]. Thus, fractures are an important public health concern.

Monitoring falls and associated fracture complications is crucial in the care of patients with schizophrenia. Compared with the general population, patients with schizophrenia present more physical problems, including osteoporosis [8]. A systematic review reported that reduced bone mass, particularly due to osteoporosis, is significantly more common in people with schizophrenia than in controls [9]. Osteoporosis is strongly correlated with fracture risk. In particular, fracture is often a consequence of osteoporosis [10]. Thus, the correlation between schizophrenia and risk of fracture is worth investigation. Considering the high influence of fractures, a small increase in fracture risk associated with schizophrenia may have a considerable public health effect. Moreover, fracture has been postulated to be correlated with cardiovascular disease [11], diabetes mellitus [12], coronary artery disease [13], cerebrovascular disease [14], and some psychiatric disorders [15, 16].

There are some studies regarding the correlation between schizophrenia and fracture [17–21]. However, most of these studies were done in western population. Furthermore, because of differences in study design, the results have been inconsistent. Most related studies were not nationwide or population-based, and some were cross-sectional studies without a cohort design. Therefore, we conducted a nationwide population-based cohort study to investigate the association between schizophrenia diagnosis and subsequent fracture. Moreover, some studies have analyzed the gender difference in osteoporosis in patients with schizophrenia but the results have been inconsistent [22–24]. Thus, we also performed a sex-based subgroup analysis to compare the risk profiles.

## Methods

### Data sources

Established in 1995, the National Health Insurance (NHI) program is a mandatory health insurance program that offers comprehensive medical care coverage, including outpatient, inpatient, emergency, and traditional Chinese medicine, to all Taiwan residents, with a coverage rate of up to 98% [25]. The NHI Research Database (NHIRD) contains comprehensive information regarding clinical visits, including prescription details and diagnostic codes, based on the A code and *International Classification of Diseases, Ninth Revision, Clinical Modification* (ICD-9-CM). The NHIRD is managed and publicly released by the National Health Research Institutes (NHRI) for research purposes, and data confidentiality is maintained according to the directives of the NHI Bureau. The data source for our study was the Longitudinal Health Insurance Database 2005 (LHID2005), which is an NHIRD data set. LHID2005 data were collected through systematic and random sampling of NHIRD data; the database includes the data of 1 million individuals. The NHRI reported that no significant differences in terms of sex distribution, age distribution, or average insured payroll-related amount between patients in the LHID2005 and those in the original NHIRD [26].

### Study population

By using data extracted from the LHID2005, we conducted a retrospective cohort study of patients newly diagnosed with schizophrenia between January 1, 2000, and December 31, 2004. Schizophrenia was defined based on ICD-9-CM code 295. To ensure diagnostic validity and patient homogeneity, we selected only patients diagnosed by psychiatrists, and least two consistent schizophrenia diagnoses were required to improve diagnostic validity. To ensure that only those who received their first diagnosis of schizophrenia between January 1, 2000, and December 31, 2004 were included in the analysis, we excluded patients diagnosed as having schizophrenia between January 1, 1996, and December 31, 1999. Furthermore, we excluded patients diagnosed with a recent hip fracture (ICD-9-CM code: 820.XX), vertebral fracture (ICD-9-CM code: 805.2, 805.4, 805.6, 805.8, 806.2, and 806.4), or wrist fracture (ICD-9-CM code: 813.4) before enrollment. For each patient with schizophrenia included in the final cohort, four age- and sex-matched controls without schizophrenia, hip fracture, vertebral fracture, or wrist fracture were randomly selected from the LHID2005. All patients with schizophrenia and controls were followed up until receipt of a diagnosis of hip, vertebral, or wrist fracture; death; withdrawal from the NHI system; or December 31, 2009. The primary clinical outcome was hip, vertebral, or wrist fracture. Furthermore, common comorbidities, including hypertension, diabetes mellitus, dyslipidemia, coronary artery disease, cerebrovascular disease, congestive heart failure, chronic pulmonary disease, osteoarthritis, and malignancy, were compared between patients with schizophrenia and controls.

### Statistical analysis

In our study, demographic features, including age, sex, typical comorbidities, urbanization, and monthly income, were compared between schizophrenia and control groups by using chi-squared and independent *t* tests. An independent *t* test was used for continuous variables and chi-squared test for nominal variables. Typical comorbidities included hypertension, diabetes mellitus, dyslipidemia, coronary artery disease, congestive heart failure, chronic lung disease, cerebrovascular disease, malignancy, and osteoarthritis. In addition, a Cox proportional-hazards regression model was constructed to calculate the hazard ratios (HRs) for hip, vertebral, and wrist fractures in the patients with schizophrenia and healthy controls. Variables such as schizophrenia diagnosis, age, sex, typical comorbidities, urbanization, and monthly income were included as covariates in the univariable model. Factors that demonstrated a moderately significant statistical relationship in the univariable analysis (*P* < .1) were entered in a multivariable Cox proportional-hazards regression model. Statistical significance was established at *P* < .05 in a multivariable Cox proportional-hazards regression model [27]. Furthermore, a sex-based subgroup analysis was performed using the Cox proportional-hazards regression model to reveal the HR of hip, vertebral, and wrist fractures in different sex groups. The interaction term between sex and schizophrenia diagnosis was added in the multivariate Cox proportional-hazards regression model to elucidate whether the differences between female patients with schizophrenia and female controls were more significant than those between male patients with schizophrenia and male controls.

SAS for Windows (version 9.3; SAS Institute, Cary, NC, USA), was used for data extraction, computation, linkage, processing, and sampling. All other statistical analyses were performed using SPSS for Windows (version 20; IBM, Armonk, NY, USA). The results with a *P* < 0.05 were considered to indicate a statistically significant difference.

## Results

The study sample comprised 2028 patients with schizophrenia and 8112 controls. The comparison of demographic and clinical variables between patients with schizophrenia and controls is presented in Table 1. The median age at the time of enrollment for patients with schizophrenia and controls was 36.3 and 36.2 years, respectively; in addition, the median (IQR) follow-up period for patients with schizophrenia and controls was 7.6 and 7.7 years, respectively. A high percentage of patients with schizophrenia were aged 20–39 years. Baseline differences in comorbidities demonstrated a high prevalence of diabetes mellitus, coronary artery disease, cerebrovascular disease, and chronic pulmonary diseases among patients with schizophrenia.

**Table 1:** Characteristics of patients with schizophrenia and control subjects

Demographic data	Patients with Schizophrenia *N* = 2028		Patients without Schizophrenia *N* = 8112		*P* values
	n	%	n	%	
Age (years) ^a^	36.3 (27.63–46.24)		36.2 (27.56–46.31)		>.99
Distribution of age					
20–39	1216	59.96	4864	59.96	
40–59	643	31.71	2572	31.71	
≥ 60	169	8.33	676	8.33	
Sex					>.99
Male	1026	50.29	4104	50.59	
Female	1002	49.41	4008	49.41	
Comorbidities					
Hypertension	232	11.44	876	10.80	.41
Diabetes mellitus	161	7.94	517	6.37	.01*
Dyslipidemia	165	8.14	687	8.47	.63
Coronary artery disease	20	0.97	22	0.27	<.01*
Congestive heart failure	26	1.28	89	1.10	.48
Cerebrovascular disease	92	4.54	173	2.13	<.01*
Chronic pulmonary disease	181	8.93	591	7.29	.01*
Osteoarthritis	185	9.12	736	9.07	.95
Malignancy	8	0.39	48	0.59	.28
Degree of urbanization					<.01*
Urban	1123	55.37	5007	61.72	
Suburban	678	33.43	2518	31.04	
Rural	227	11.19	587	7.24	
Income group					<.01*
Low income	1449	71.45	3298	40.66	
Medium income	532	26.23	3264	40.24	
High income	47	2.32	1550	19.11	
Follow-up, years ^a^	7.60 (6.21–9.02)		7.72 (6.42–9.03)		<.01*
Newly diagnosed fracture, N (%)	89	4.39	257	3.17	.01*
Hip fracture	25	1.23	54	0.67	.01*
Vertebral fracture	53	2.61	142	1.75	.01*
Wrist fracture	18	0.89	76	0.94	.84

^a^Median (interquartile range); * Statistical significance

During the follow-up period, 89 patients with schizophrenia (4.4%) and 257 controls (3.2%) were diagnosed with hip, vertebral, or wrist fracture (*P* = 0.007). Among the patients with schizophrenia, 25 (1.2%), 53 (2.6%), and 18 (0.9%) patients were diagnosed with hip, vertebral, and wrist fractures, respectively. Overall, the incidences of hip (*P* = 0.009) and vertebral (*P* = 0.011) fractures were significantly higher in patients with schizophrenia than in controls.

In addition, a Cox proportional-hazards regression analysis was conducted to calculate the HRs of newly diagnosed hip, vertebral, and wrist fractures among patients with schizophrenia relative to the matched controls (Tables 2–4). The results indicated that patients with schizophrenia have a markedly higher risk of hip fracture (HR: 1.78) and vertebral fracture (HR: 1.40) but not of wrist fracture.

**Table 2:** Analyses of risk factors for hip fracture in patients with and without schizophrenia

Predictive variables	Univariate analysis		Multivariate analysis	
	HR (95% CI)	*P* value	HR (95% CI)	*P* value
Schizophrenia	1.89 (1.18–3.04)	.01	1.78 (1.08–2.93)	.02*
Age (< 60 = 0, ≥60 = 1)	19.02 (12.07–29.97)	<.01	10.85 (6.09–19.33)	<.01*
Sex (Female = 1, Male = 0)	0.93 (0.60–1.15)	.76		
Comorbidities				
Hypertension	7.81 (5.02–12.16)	<.01	1.81 (1.03–3.20)	.04*
Diabetes mellitus	4.77 (2.85–8.00)	<.01	1.27 (0.70–2.30)	.43
Dyslipidemia	3.25 (1.90–5.56)	<.01	0.81 (0.44–1.51)	.51
Coronary artery disease	10.82 (3.41–34.3)	<.01	1.79 (0.53–6.02)	.35
Congestive heart failure	9.41 (4.33–20.46)	<.01	1.37 (0.59–3.19)	.46
Cerebrovascular disease	6.75 (3.57–12.78)	<.01	1.20 (0.60–2.41)	.61
Chronic pulmonary disease	3.93 (2.32–6.66)	<.01	1.03 (0.57–1.85)	.94
Osteoarthritis	4.81 (2.98–7.78)	<.01	1.48 (0.87–2.53)	.15
Malignancy	5.40 (1.33–21.98)	.02	1.63 (0.39–6.79)	.50
Degree of urbanization				
Urban	Reference		Reference	
Suburban	1.52 (0.95–2.43)	.08	1.42 (0.88–2.29)	.16
Rural	1.74 (0.85–3.60)	.13	1.32 (0.62–2.80)	.47
Income group				
Low income	Reference		Reference	
Medium income	0.40 (0.23–0.68)	<.01	0.58 (0.33–1.01)	.05
High income	0.26 (0.11–0.66)	<.01	0.89 (0.33–2.37)	.81

HR indicates hazard ratio; CI indicates confidence interval

**Table 3:** Analyses of risk factors for vertebral fracture in patients with and without schizophrenia

Predictive variables	Univariate analysis		Multivariate analysis	
	HR (95% CI)	*P* value	HR (95% CI)	*P* value
Schizophrenia	1.53 (1.12–2.10)	.01	1.40 (1.01–1.95)	.05*
Age (< 60 = 0, ≥60 = 1)	6.09 (4.53–8.18)	<.01	2.89 (1.98–4.21)	<.01*
Sex (Female = 1, Male = 0)	2.18 (1.61–2.95)	<.01	1.80 (1.33–2.44)	<.01*
Comorbidities				
Hypertension	4.55 (3.38–6.13)	<.01	1.89 (1.28–2.79)	<.01*
Diabetes mellitus	3.78 (2.66–5.37)	<.01	1.47 (0.96–2.24)	.07
Dyslipidemia	2.68 (1.87–3.85)	<.01	0.89 (0.58–1.36)	.59
Coronary artery disease	12.18 (6.00–24.73)	<.01	3.62 (1.71–7.67)	<.01*
Congestive heart failure	5.79 (3.15–10.64)	<.01	1.06 (0.54–2.09)	.86
Cerebrovascular disease	2.49 (1.35–4.57)	.01	0.65 (0.34–1.24)	.19
Chronic pulmonary disease	3.03 (2.11–4.35)	<.01	1.33 (0.89–1.99)	.17
Osteoarthritis	3.80 (2.76–5.25)	<.01	1.71 (1.18–2.48)	<.01*
Malignancy	2.09 (0.52–8.41)	.30		
Degree of urbanization				
Urban	Reference			
Suburban	1.14 (0.84–1.55)	.41		
Rural	1.47 (0.92–2.34)	.11		
Income group				
Low income	Reference		Reference	
Medium income	0.70 (0.52–0.95)	.02	0.88 (0.64–1.20)	.41
High income	0.30 (0.17–0.55)	<.01	0.56 (0.30–1.03)	.06

HR indicates hazard ratio; CI indicates confidence interval

**Table 4:** Analyses of risk factors for wrist fracture in patients with and without schizophrenia

Predictive variables	Univariate analysis		Multivariate analysis	
	HR (95% CI)	*P* value	HR (95% CI)	*P* value
Schizophrenia	0.96 (0.58–1.61)	.89	0.96 (0.57–1.61)	.88
Age (< 60 = 0, ≥60 = 1)	4.61 (2.95–7.21)	<.01	3.51 (2.01–6.13)	<.01*
Sex (Female = 1, Male = 0)	1.49 (0.99–2.25)	.06	1.37 (0.90–2.08)	.14
Comorbidities				
Hypertension	2.52 (1.55–4.09)	<.01	1.11 (0.60–2.07)	.74
Diabetes mellitus	1.97 (1.05–3.70)	.04	0.88 (0.42–1.82)	.72
Dyslipidemia	2.41 (1.41–4.14)	<.01	1.45 (0.76–2.77)	.26
Coronary artery disease	0.05 (0.01–111,081)	.68		
Congestive heart failure	3.10 (0.98–9.79)	.05	0.92 (0.27–3.14)	.90
Cerebrovascular disease	2.81 (1.23–6.43)	.01	1.24 (0.51–3.02)	.64
Chronic pulmonary disease	2.50 (1.44–4.34)	<.01	1.48 (0.80–2.72)	.21
Osteoarthritis	1.90 (1.08–3.36)	.03	0.92 (0.49–1.73)	.79
Malignancy	2.14 (0.30–15.32)	.45		
Degree of urbanization				
Urban	Reference			
Suburban	1.39 (0.90–2.15)	.14	1.36 (0.88–2.10)	.17
Rural	1.74 (0.91–3.36)	.10	1.46 (0.75–2.82)	.27
Income group				
Low income	Reference			
Medium income	0.91 (0.59–1.40)	.66		
High income	0.68 (0.35–1.30)	.24		

HR indicates hazard ratio; CI indicates confidence interval

Furthermore, a sex-based subgroup analysis revealed that the risk of hip fracture was significantly higher in female patients with schizophrenia than in female controls (HR: 2.68); however, the risk of hip fracture did not differ significantly between male patients with schizophrenia and male controls. Moreover, for both male and female subgroups, the risks of vertebral and wrist fractures did not differ significantly between patients with schizophrenia and controls. After including the interaction term between sex and schizophrenia diagnosis in the model, no effect of the association between sex and schizophrenia diagnosis was observed on the risk of fractures (Table 5).

**Table 5:** Interaction between sex and schizophrenia on the risk of fractures

	Adjusted HR of male cohort (95% CI)	Adjusted HR of female cohort (95% CI)^a^	*P*-value for comparison
Hip fracture	1.27 (0.60–2.65)	2.68 (1.32–5.44) *	.20
Vertebral fracture	1.62 (0.91–1.04)	1.29 (0.86–1.94)	.55
Wrist fracture	0.65 (0.26–1.62)	1.25 (0.65–2.43)	.64

*HR* Hazard ratio, *CI* Confidence interval; * Statistical significance

^a^ Adjusted for age, hypertension, diabetes mellitus, dyslipidemia, coronary artery disease, cerebrovascular disease, congestive heart failure, chronic pulmonary disease, osteoarthritis, malignancy, urbanization and income

## Discussion

This is one of the few representative clinical cohort studies examining the effect of schizophrenia on the risk of site-specific skeletal fractures by using a nationwide population-based data set over a 10-year follow-up period. The major finding of this study was the significantly high incidence and risk of hip and vertebral fractures among patients with schizophrenia compared with controls.

As shown in Table 1, the patients with schizophrenia had more comorbidities than the controls including diabetes, coronary heart disease, cerebrovascular disease, chronic obstructive pulmonary disease, and osteoporosis. This is consistent with the findings of previous reports [8, 28]. As shown in Tables 2, 3, 4, after adjusting for the confounding factors, the HRs for hip and vertebral fractures were 1.78 and 1.40, respectively, and both values were significant. These results suggested that the schizophrenia cohort had significantly high risks of hip and vertebral fractures compared with the control cohort. Furthermore, Cox proportional-hazards regression analysis revealed that, in addition to schizophrenia diagnosis, other risk factors for fractures include age; sex; and the comorbidities of hypertension, coronary artery disease, and osteoarthritis. These results are compatible with those in previous reports [13, 29, 30].

Few studies have explored the correlation between schizophrenia and fractures, and they have reported inconsistent results. Bolton et al. revealed that schizophrenia is associated with fractures (odds ratio [OR]: 2.17, 95% confidence interval [CI]: 1.75–2.69) [21]. Moreover, Howard et al. reported that hip fracture is associated with both schizophrenia (OR: 1.73; 95% CI: 1.32–2.28) and the use of prolactin-raising antipsychotics (OR: 2.6; 95% CI: 2.43–2.78); however, in their multivariate analysis, schizophrenia diagnosis did not have an independent association with fracture risk [20]. Sorensen et al. conducted a national cohort study and found that, after adjusting for the established risk factors for fracture, schizophrenia diagnosis was associated with an increased risk of hip fracture, but the effect was nonsignificant after adjustment for psychotropics [19]. In a case–control study using data from the Taiwan NHIRD, Wu et al. reported a similar result that current antipsychotic use is associated with an increased risk of hip fracture in patients with schizophrenia [31]. However, Tsai et al. conducted a nationwide cohort study based on data from the same Taiwan NHIRD, which revealed that the proportion of days with psychiatric medication does not have a dose–response relationship with fracture risk [32]. Bishop et al. conducted a study in female patients and concluded that fractures in the past 12 months were more likely in the schizophrenia cohort than in the control cohort [17]; Kelly et al. reported similar results in the schizophrenia and control groups [18]. In a comparative meta-analysis of eight studies, Stubbs et al. revealed that patients with schizophrenia have a higher fracture risk compared with the control population (incidence rate ratio: 1.72; 95% CI: 1.24–2.39) [33]. The present study revealed similar findings and further analyzed the risk of fractures at different sites.

The possible explanations for increased fracture risk among patients with schizophrenia are multifactorial and may include (1) the influence of psychotropic medications, (2) schizophrenia-related poor lifestyle and substance use, and (3) some common schizophrenia comorbidities. First, the short-term side effects of antipsychotics, antidepressants, and benzodiazepines, which include sedation, extrapyramidal symptoms, somnolence, and orthostatic hypotension, may affect patient balance and gait, leading to an increased risk of fall and fracture [34–36]. Some studies have investigated the influence of prolactin-increasing antipsychotics on the bone mineral density of patients with schizophrenia. In theory, hyperprolactinemia is associated with decreased bone mineral density, which may increase fracture risk [37, 38]. Sustained hyperprolactinemia may accelerate bone turnover and increase bone resorption more than bone formation, contributing to an osteopenic effect mediated by the hypothalamic–pituitary–gonadal axis [39–41]. However, no study has reported a consistent association between hyperprolactinemia and reduced bone mineral density in patients with schizophrenia [42].

Second, sedentary lifestyle, staying indoors, and poor nutritional intake are frequently observed in patients with schizophrenia, and these behaviors contribute to reduced muscle strength, low sun exposure, and vitamin D deficiency, followed by reduced bone mineral density and increased fall and fracture risk [43, 44]. Studies have demonstrated that low physical activity in patients with schizophrenia is significantly correlated with negative symptoms [45]. Muscle weakness has been reported to be a potential key predictor for falls and fractures [46], and muscular fitness is impaired in patients with schizophrenia, which may contribute to their reduced walking capacity [47]. Therefore, a multidisciplinary team, including physiotherapists and exercise physiologists, may aid in reducing fracture risk in patients with schizophrenia [48]. Furthermore, smoking and alcohol use are common in patients with schizophrenia, both of which are established risk factors for osteoporosis and osteoporotic fracture [49, 50].

Third, certain comorbidities of schizophrenia, including diabetes mellitus, contribute to fracture risk. Patients with schizophrenia have an increased risk of diabetes mellitus, which is the key risk factor for fractures in the general population [51].

Some studies have analyzed the gender difference in osteoporosis in patients with schizophrenia but the results have been inconsistent. Renn et al. [22] indicated that the prevalence of severe low bone mass was much higher for female schizophrenic patients than male patients in all the age groups, whereas Hummer et al. [23] concluded that in men but not women with schizophrenia, bone mineral density was significantly lower than normal in the lumbar region. In a study of schizophrenia patients aged 50 or older, Jung et al. [24] found a significant gender difference, with 48.4% of female patients showing osteopenia or osteoporosis compared with 25.7% male patients (*P* = 0.0014). In the present study, a sex-based subgroup analysis revealed that the risk of hip fracture remained significantly higher in female patients with schizophrenia than in the female controls. One explanation is that menopause-induced estrogen deficiency, presents only in female, has been proved to be a major risk factor for the loss of bone mineral density which increases the risk of osteoporosis and fractures among perimenopausal and postmenopausal women [52]. In female patients of schizophrenia, the prolactin-increasing antipsychotics may contribute to menopause and hyperprolactinemia which are associated with decreased bone mineral density and furtherly increase fracture risk [37, 38]. However, after inclusion of the interaction term between sex and schizophrenia in the model, no effect of the association between sex and schizophrenia on the risk of fractures was observed. Considering differences in the designs of the abovementioned studies, a more comprehensive investigation is required to determine the correlation between sex and fracture risk among patients with schizophrenia.

The strengths of this study are discussed as follows. First, this was a nationwide representative study. Second, this was a cohort study with an average follow-up of 8 years. Third, the study used data from the NHIRD, which enabled a comparison of a range of relevant clinical variables between patients with schizophrenia and controls. Fourth, the study included three fracture sites for a more comprehensive analysis. Fifth, we considered both sexes and performed a sex-specific analysis. Therefore, the results of this nationwide population-based cohort study on the influence of fractures on patients with schizophrenia may provide useful contributions to the literature.

Several limitations of this study, which are inherent to claims database, must be considered. First, the causes of fractures are usually complex and vary depending on the individual. The potential risk factors of osteoporotic fractures include age, sex, body mass index, tobacco use, alcohol consumption, bone mineral density, physical inactivity, and depression [53–55]. Many psychological and environmental factors can also contribute to fracture risk [56]. Several important demographic variables, such as alcohol consumption, tobacco use, lifestyle, body mass index, and physical activity, are unavailable in the NHIRD. Second, psychotropic drugs have been suggested to be an important confounder that increases the incidence of fracture [57–59]. However, we did not include data related to psychotropics in this study because of the complexity of pharmacological prescriptions, such as polypharmacy, frequent switching of psychotropics, self-medication, and the high possibility of poor drug adherence, in patients with schizophrenia. This limitation is unavoidable in a retrospective study based on NHIRD data. Further prospective studies that take into account psychotropics are required. Third, the follow-up duration in our study may have been insufficient for detecting late onset fractures. Thus, future studies with longer follow-up periods are required to more thoroughly elucidate the long-term risk of fractures in patients with schizophrenia. Fourth, the sample size of some subgroups was small, especially the subgroup of patients with schizophrenia with wrist fractures (*n* = 18), which may have contributed to a low statistical power in these groups. Fifth, some mental health problems other than schizophrenia have also been reported to increase the risk of fracture. However, while the common mental health problems such as the depression may also exist in schizophrenia patients, those with the pure diagnosis of schizophrenia shouldn’t comorbid with other major mood disorders making the control of mood symptoms impossible. These confounding factors should be acknowledged.” Given the scarcity of cohort studies on this topic, additional studies must be conducted to obtain evidence clarifying the association between schizophrenia and fractures.

## Conclusion

In conclusion, this nationwide cohort study revealed increased risks of hip and vertebral fractures in patients with schizophrenia during a 10-year follow-up. Hip fracture risk was significantly higher in female patients with schizophrenia than in female controls. The findings are of great clinical implications which remind the clinicians to increase the awareness of fracture risk when managing patients with schizophrenia. Considering the high morbidity and mortality in patients with schizophrenia who experienced a fracture, strategies must be developed to improve lifestyle and bone health, which may reduce the possibility of subsequent fractures. To confirm our findings, prospective studies, particularly those with additional patient-level data, are warranted.

## Data Availability

The data that support the findings of this study are available from the Taiwan National Health Insurance Research Database (NHIRD). Interested individuals should contact the NHRI to gain access (https://dep.mohw.gov.tw/DOS/cp-5119-59201-113.html).

## Abbreviations

CI: Confidence interval
HR: Hazard ratio
ICD-9-CM: International Classification of Diseases, Ninth Revision, Clinical Modification
IQR: Interquartile range
LHID 2005: Longitudinal Health Insurance Database 2005
NHI: National Health Insurance
NHIR: National Health Research Institutes
NHIRD: NHI Research Database
OR: Odds ratio
